# Free essential amino acid feeding improves endurance during resistance training via DRP1‐dependent mitochondrial remodelling

**DOI:** 10.1002/jcsm.13519

**Published:** 2024-06-16

**Authors:** Jiwoong Jang, Yeongmin Kim, Taejeong Song, Sanghee Park, Hee‐Joo Kim, Jin‐ho Koh, Yoonil Cho, Shi‐Young Park, Sakthivel Sadayappan, Hyo‐Bum Kwak, Robert R. Wolfe, Il‐Young Kim, Cheol Soo Choi

**Affiliations:** ^1^ Integrative Metabolic Fluxomics Lab, Lee Gil Ya Cancer and Diabetes Institute Gachon University Incheon Korea; ^2^ Korea Mouse Metabolic Phenotyping Center, Lee Gil Ya Cancer and Diabetes Institute Gachon University Incheon Korea; ^3^ Department of Internal Medicine, Gil Medical Center Gachon University Incheon Korea; ^4^ Department of Health Sciences and Technology, GAIHST Gachon University Incheon Korea; ^5^ Department of Internal Medicine, Division of Cardiovascular Health and Disease, Center for Cardiovascular Research University of Cincinnati Cincinnati Ohio USA; ^6^ Department of Molecular Medicine, College of Medicine Gachon University Incheon Korea; ^7^ Gachon Biomedical Convergence Institute Gachon University Gil Medical Center Incheon Korea; ^8^ Department of Kinesiology Inha University Incheon Korea; ^9^ Institute of Sports & Arts Convergence Inha University Incheon Korea; ^10^ Department of Biomedical Science, Program in Biomedical Science & Engineering Inha University Incheon Korea; ^11^ Department of Geriatrics, Center for Translational Research in Aging and Longevity, Donald W. Reynolds Institute on Aging University of Arkansas for Medical Sciences Little Rock Arkansas USA

**Keywords:** Metabolic flux, Mitochondrial dynamics, Muscle mass, Physical performance, Protein synthesis rate, Neuromuscular junction stability

## Abstract

**Background:**

Loss of muscle strength and endurance with aging or in various conditions negatively affects quality of life. Resistance exercise training (RET) is the most powerful means to improve muscle mass and strength, but it does not generally lead to improvements in endurance capacity. Free essential amino acids (EAAs) act as precursors and stimuli for synthesis of both mitochondrial and myofibrillar proteins that could potentially confer endurance and strength gains. Thus, we hypothesized that daily consumption of a dietary supplement of nine free EAAs with RET improves endurance in addition to the strength gains by RET.

**Methods:**

Male C57BL6J mice (9 weeks old) were assigned to control (CON), EAA, RET (ladder climbing, 3 times a week), or combined treatment of EAA and RET (EAA + RET) groups. Physical functions focusing on strength or endurance were assessed before and after the interventions. Several analyses were performed to gain better insight into the mechanisms by which muscle function was improved. We determined cumulative rates of myofibrillar and mitochondrial protein synthesis using ^2^H_2_O labelling and mass spectrometry; assessed ex vivo contractile properties and in vitro mitochondrial function, evaluated neuromuscular junction (NMJ) stability, and assessed implicated molecular singling pathways. Furthermore, whole‐body and muscle insulin sensitivity along with glucose metabolism, were evaluated using a hyperinsulinaemic–euglycaemic clamp.

**Results:**

EAA + RET increased muscle mass (10%, *P* < 0.05) and strength (6%, *P* < 0.05) more than RET alone, due to an enhanced rate of integrated muscle protein synthesis (19%, *P* < 0.05) with concomitant activation of Akt1/mTORC1 signalling. Muscle quality (muscle strength normalized to mass) was improved by RET (i.e., RET and EAA + RET) compared with sedentary groups (10%, *P* < 0.05), which was associated with increased AchR cluster size and MuSK activation (*P* < 0.05). EAA + RET also increased endurance capacity more than RET alone (26%, *P* < 0.05) by increasing both mitochondrial protein synthesis (53%, *P* < 0.05) and DRP1 activation (*P* < 0.05). Maximal respiratory capacity increased (*P* < 0.05) through activation of the mTORC1‐DRP1 signalling axis. These favourable effects were accompanied by an improvement in basal glucose metabolism (i.e., blood glucose concentrations and endogenous glucose production vs. CON, *P* < 0.05).

**Conclusions:**

Combined treatment with balanced free EAAs and RET may effectively promote endurance capacity as well as muscle strength through increased muscle protein synthesis, improved NMJ stability, and enhanced mitochondrial dynamics via mTORC1‐DRP1 axis activation, ultimately leading to improved basal glucose metabolism.

## Introduction

Muscle strength and endurance affect one's health and quality of life, particularly in aging. Muscle strength is largely determined by the (protein) mass of skeletal muscle. In addition, muscle serves as the largest amino acid reservoir in the body that provides building blocks to other organs for making new proteins for virtually every body function. Thus, loss of muscle mass and strength negatively affects health and contributes to other clinical conditions such as cancer, cardiovascular diseases, diabetes, and obesity.^1^ While cardiorespiratory fitness (CRF), determined as endurance, is also a determinant in predicting health,^2^ resistance exercise training (RET) alone does not typically improve both muscle strength and CRF concurrently.^3^ Therefore, it is desirable and important to discover a practical approach to improving muscle mass and function as well as CRF.

Supplementation of the diet with a balanced composition of free essential amino acids (EAAs) may enhance both muscle strength and endurance during RET, as EAAs stimulate synthesis of contractile and mitochondrial proteins.^4^ It is well demonstrated that RET stimulates muscle protein synthesis (MPS), and the anabolic effect of RET is synergistically amplified with the provision of EAAs.^4^ Although less well established, EAAs also have an anabolic effect on mitochondrial protein synthesis. For example, it was shown that supplementation of a balanced free EAA supplement for 3 months improved endurance in sedentary mice by increasing mitochondrial abundance.^5^

Endurance capacity is largely influenced by mitochondrial volume and function.^6^ In this regard, mitochondrial dynamics (i.e., fission and fusion as well as protein homeostasis) play an essential role in maintaining the function and abundance of mitochondria.^7^ Protein turnover replaces the old, poorly functioning proteins of mitochondria with new, better‐functioning proteins. Dynamin‐related protein 1 (DRP1) and Dynamin‐like 120 kDa protein (OPA1) are involved in fission and fusion processes, respectively. It has been shown that EAAs increase mitochondrial biogenesis and thus endurance.^5^ Furthermore, it is well established that leucine, one of nine EAAs,^8^ activates mammalian target of rapamycin complex 1 (mTORC1), a known upstream regulator of DRP1.^9^ However, no studies have directly demonstrated that EAA supplementation affects mitochondrial dynamics via modulating DRP1 and/or OPA1.

Whereas it has been demonstrated that free EAAs amplify adaptations of RET (i.e., anabolic response) and stimulate muscle mitochondrial biogenesis,^4^, ^5^ it remains unclear whether EAA supplementation can improve endurance during RET as well. Therefore, in the present study, we investigated whether the supplementation of the normal diet with free EAAs during RET can enhance both endurance and strength as compared with RET alone. We further aimed to elucidate the underlying mechanisms associated with the enhanced responses.

## Methods

### Animal care and experiment design

Nine‐week‐old C57BL/6J male mice were randomly assigned to four groups: control (CON) essential amino acids (EAA), resistance exercise training (RET), and EAA with RET (EAA + RET). EAAs were orally administered twice a day (1.5 g/kg/day) at 9 am and 5 pm, and on days with exercise, first bolus was administered immediately after exercise. To differentiate chronic effects from acute effects, muscle samples from two mice cohorts were collected 1 h or 72 h after the last respective treatment, respectively. The detailed composition of EAAs is presented in Table S1.

### Resistance exercise training protocol

Climbing of a 1 m ladder with a 1.5 cm grid inclining at 85° was used as resistance exercise training, as previously described.^10^ Briefly, during each session, mice climbed the ladder with 50%, 70%, 90%, and 100% of the previous maximal carrying weight, and the load was increased by 3 g for each subsequent repetition up to 10 repetitions. Once the mice reached the top of the ladder, they rested for 2 min.

### Physical function tests

Maximum carrying capacity (MCC) and four‐limb grip strength for strength and treadmill running for endurance capacity were measured to determine physical function after 4 weeks of interventions, as previously described.^4^ In all groups, MCC was measured on the morning of the last intervention day, and four‐limb grip strength and endurance were measured 48 h after the MCC measurements.

### Ex vivo muscle force measurement

To identify whether changes in muscle quality was due to changes in intramuscular factors, ex vivo muscle contractile property were measured as previously described.^11^ Briefly, extensor digitorum longus (EDL) was skinned in a glycerinated relaxing solution at 4°C. Aluminium T‐clips were attached to the skinned single muscle fibre ends and mounted between the force transducer and the length controller (403A and 322C, respectively: Aurora Scientific, ON, Canada). While the fibre was visualized under the microscope, the sarcomere length was adjusted to the initial length (L_0_, 2.3 μm) using HeNe laser diffraction, and the fibre diameter was calculated manually. Next, isometric force of each muscle fibre was measured at various Ca^2+^ concentrations ranging from pCa 9.0 to 4.5. The absolute force was normalized to the fibre cross‐sectional area (CSA) assuming a cylindrical shape.

### Immunofluorescence

To measure myofiber CSA, sections were incubated overnight at 4°C with rabbit anti‐laminin. After washing muscle sections were incubated with Alexa Fluor 488‐conjugated anti‐rabbit IgG for 1 h. Images were acquired using a confocal microscope (Zeiss LSM 700, Oberkochen, Germany).

To measure acetylcholine receptor (AchR) size plantaris s were fixed at 4% paraformaldehyde in PBS. After washing muscles were incubated overnight at 4°C with the primary Alexa Fluor 488‐conjugated anti‐rabbit α‐Bungarotoxin. The muscles were then incubated with Alexa Fluor 488‐conjugated anti‐rabbit IgG for 1 h at RT. ImageJ (National Institutes of Health) image analysis software was used for fibre CSA and AchR cluster calculations. The antibodies information was presented in Table S2.

### Isolation of myofibrillar and mitochondrial proteins

The Differential centrifugation method was used to separate myofibrils and mitochondrial proteins.^12^ Briefly, gastrocnemius was homogenized in buffer (550 mM KCl, 5 mM EGTA, and 100 mM MOPS) and centrifuged at 800 *g* for 5 min at 4°C to collect myofibril fractions. Thereafter, the mitochondrial fraction was collected by further centrifugation at 7000 *g* for 10 min at 4°C.

### Heavy water labelling and calculations of muscle protein kinetics

To analyse cumulative rates of MPS, an intraperitoneal bolus of 35 ml/kg of 99% ^2^H_2_O (DLM‐4, Cambridge isotope laboratories, MA, USA) was given with 0.9% NaCl, followed by ad libitum access to drinking water enriched to 8% ^2^H_2_O for the last 2 weeks of the intervention. The calculation of muscle protein fractional synthesis rate (FSR, %/time) was predicated on the precursor‐product rule.^13^, ^14^ The precursor enrichment was inferred from the ^2^H enrichment of body fluid (e.g., plasma) accounting for ~3.7 potential ^2^H exchange sites of alanine.^15^

$$\mathrm{FSR}\;\left(\%/t\right)=\left[\left(E_{\mathrm{Ala}}\right)/\left(E_{\mathrm{BW}}\times 3.7\right)\right]\times 100.$$

$$k_{s}=-\ln\;\left(1-\mathrm{FSR}\right)/t.$$

$$\text{Absolute synthesis}\;\left(\mathrm{mg}/\mathrm{day}\times 14\;\text{days}\right)=k_{s}\left({\mathrm{day}}^{-1}\right)\times \text{muscle pool size}\;\left(\mathrm{mg}\right)\times 14\;\text{days}.$$

where enrichment (E) is expressed as mole percent excess (MPE) calculated as a tracer to tracee ratio; t is time of labelling; and *ks* represents fractional synthesis rate constant.^16^ The absolute synthesis rate of muscle protein was calculated by multiplying the cumulative *ks* over the 14 days by muscle protein pool size with the assumption of 12% of total muscle wet weight being myofibrillar protein in muscle.^17^

### Immunoblot analysis

Immunoblot analysis was performed using antibodies in Table S2 to identify implicated molecular signalling as previously described.^18^ In all analyses, β‐tubulin was used as a loading control, and OPA1 was quantified together without distinguishing between the two bands.^19^, ^20^ Immunodetection was carried out with ECL detection reagent (GE Healthcare, Buckingham, UK). The Protein amount was analysed using ImageJ software.

### Mitochondrial DNA copy number quantification and RT PCR

Mitochondrial DNA copy number was determined to investigate relationship between endurance capacity and mitochondrial volume as previously described.^4^ Briefly, DNA was extracted from gastrocnemius using QIAmp DNA minikit (Qiagen, Hilde, Germany). Then, 25 ng of total DNA was amplified with TOPrealTM qPCR premix (Enzynimics, Daejeon, Republic of Korea). The primer sequence for the mtDNA (*mtMDA4*), and internal control (*28S rRNA*) is presented in Table S3. RT‐PCR analysis was performed as described previously^18^ and the primer sequence for *Atrogin‐1*, *MuRF1*, and internal control (*GAPDH*) is presented in Table S3.

### Cell culture and small interfering RNA transfections

To investigate whether EAAs changed mitochondrial dynamics in a DRP1‐dependent manner, cultured C2C12 myotubes were transfected with 40 nM of DRP1 siRNA in Opti‐mem (Thermo Fisher Scientific, MA, USA), using Lipofectamine 2000 (Invitrogen, CA, USA) according to the manufacturer's instructions. The target sequence for siDRP1 and control siRNA were presented in Table S4. Thereafter, the culture medium was switched into DMEM supplemented 2% horse serum, for differentiation into myotubes. Total proteins were collected 5 days after transfection for further analysis.

### Mitochondrial function test

C2C12 cells were cultured in Seahorse XFe24 plates to assess mitochondrial function. One hour prior to perfume the mitochondrial stress test, the medium was changed to XF base medium (Agilent, CA, USA) supplemented with glucose, glutamine, and sodium bicarbonate and allowed to equilibrate in a non‐CO_2_ incubator for 1 h. After baseline measurement, 1.5 mM oligomycin, 1.0 mM carbonyl cyanide 4‐(trifluoromethoxy)phenylhydrazone, and a combination of 0.5 mM rotenone and antimycin A was sequentially injected to measure basal respiration, reserve capacity, and maximal respiratory capacity using a Seahorse Extracellular Flux Analyser (Agilent, CA, USA).

### Hyperinsulinaemic–euglycaemic clamp

Hyperinsulinaemic–euglycaemic (H‐E) clamps was performed to assess basal glucose metabolism and insulin sensitivity as modified from the previously described method.^21^ After an overnight fast, [3‐^3^H]glucose (PerkinElmer, MA, USA) was infused at a rate of 0.05 μCi/min for 2 h to assess the rate of basal glucose turnover. Followed by the basal period, H‐E clamp was conducted for 150 min with a primed/continuous infusion of human insulin (7.15 mU/kg prime and at a rate of 3.0 mU/kg/min infusion) while plasma glucose was maintained at basal concentrations (~130 mg/dL). Throughout the clamp, [3‐^3^H]‐glucose was infused at a rate of 0.1 μCi/min to estimate insulin‐stimulated whole‐body glucose fluxes, and 2‐deoxy‐D‐[1‐^14^C]glucose (PerkinElmer, MA, USA) was injected as a bolus at the 125th minute of the clamp to estimate the rate of insulin‐stimulated tissue glucose uptake. Blood samples were taken at the end of the basal period and during the last 60 min of the clamp for the measurement of specific activity of plasma ^3^H and ^14^C.

### Calculations of whole‐body and muscle glucose kinetics

To determine plasma ^3^H and ^14^C specific activities, plasma was deproteinized with Ba(OH)_2_ and ZnSO4, dried to remove ^3^H_2_O, resuspended in water, and counted in scintillation fluid (Ultima gold, PerkinElmer, MA, USA). The rates of basal and insulin‐stimulated whole‐body glucose flux were determined as the ratio of the [3‐^3^H]‐glucose infusion rate (disintegrations per minute, DPM) to the specific activity of plasma glucose (DPM per mg) at the end of the basal period and during the 30 min from 90th to 120th min of the clamp experiment, respectively. Hepatic glucose production (HGP) was determined by subtracting the glucose infusion rate from the total glucose rate of appearance. The plasma‐specific activity of ^3^H_2_O was determined by the difference between ^3^H counts with and without drying. Whole‐body glycolysis rate was calculated from the rate of the increase in specific activity of plasma ^3^H_2_O divided by that of plasma ^3^H‐glucose, as previously described.^22^ Whole‐body glycogen synthesis rate was estimated by subtracting whole‐body glycolysis from whole‐body glucose uptake, with the assumption that rates of glycolysis and glycogen synthesis account for the majority of insulin‐stimulated glucose uptake.^23^ To determine individual tissue glucose uptake, tissue samples were homogenized, and the supernatants were subjected to an ion‐exchange column to separate tissue ^14^C‐2‐DG‐6‐phosphate (2‐DG‐6‐P) from 2‐DG. Tissue glucose uptake was calculated from the area under the curve of plasma ^14^C‐2‐DG profile for the last 25 min of the clamp and muscle ^14^C‐2‐DG‐6‐P content, as previously described.^22^

### Statistical analysis

One‐way analysis of variance (ANOVA) was performed to validate intervention groups on variables, and Fisher's least significant difference (LSD) post hoc test was used for multiple comparisons. Statistical significance was set at *P* < 0.05. All data were expressed as mean ± SE.

## Results

### Essential amino acid feeding improves endurance capacity and amplifies resistance exercise training adaptations

**Figure 1:**
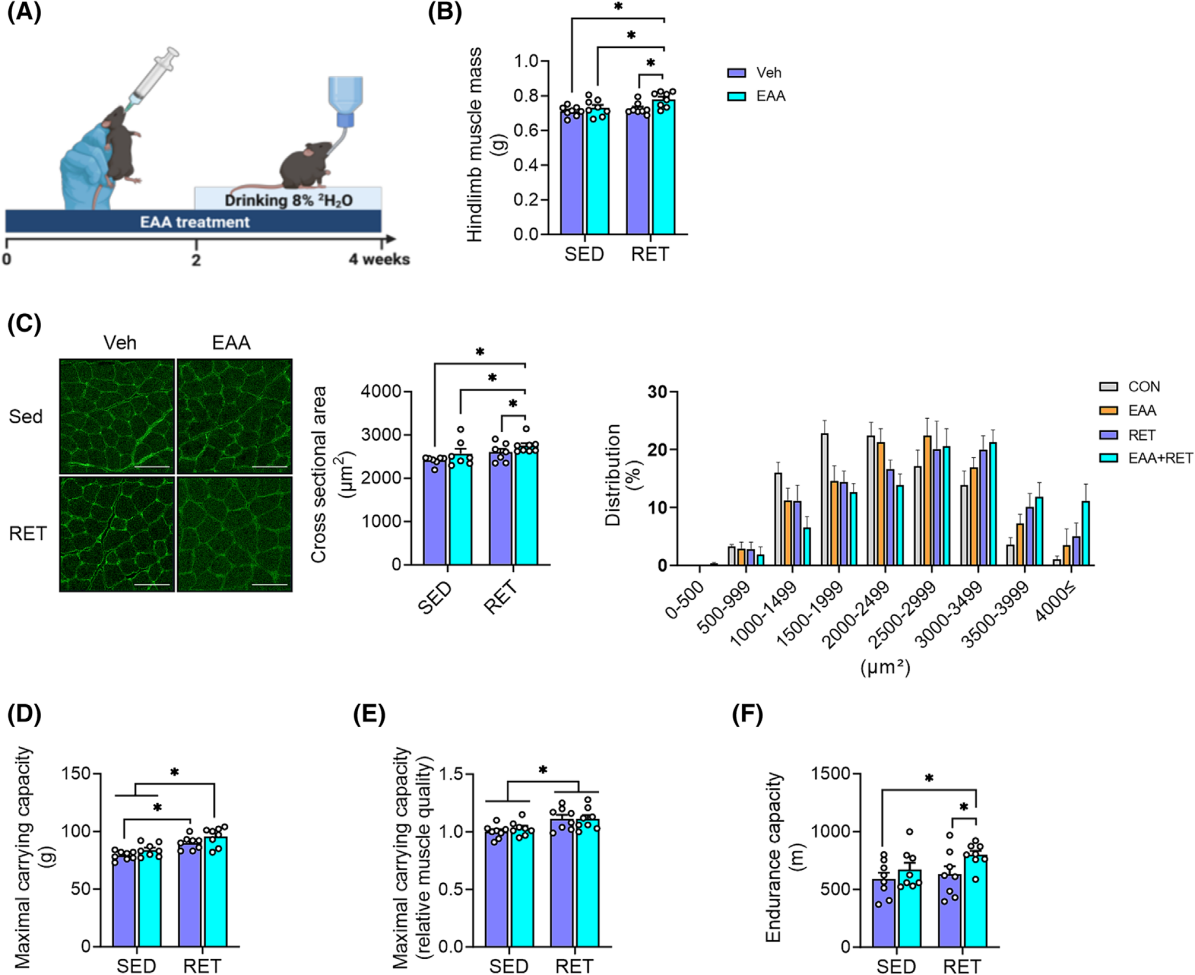
EAA + RET increases not only muscle mass and strength but endurance capacity. (A) Schematic representation of the experimental design. This figure was created with BioRender.com. (B) Total hindlimb muscle mass (sum of soleus, plantaris, flexor hallucis longus, gastrocnemius, extensor digitorum longus, and tibialis anterior weight) (*n* = 8 per group). (C) Laminin staining of muscle cross‐sectional area and distribution of myofiber size in gastrocnemius (*n* = 7–8 per group). Scale bar, 100 μm. (D) Muscle strength is representative of maximal carrying capacity (*n* = 8 per group). (E) Relative muscle quality in maximal carrying capacity (maximal carrying capacity normalized by hindlimb muscle mass). (F) Total running distance during the treadmill exhaustion test. Data are presented as mean ± SE. *Significant difference between labelled groups (*P < 0.05). EAA + RET, essential amino acids + resistance exercise training; EAA, essential amino acids; RET, resistance exercise training; SED, sedentary; Veh, vehicle.

After 4 weeks of intervention, EAA + RET increased both absolute and relative muscle mass and CSA compared with other groups (Figure 1B,C) without changes in body weight or food intake (Figure S1A–C). RET‐only group increased relative, but not absolute, muscle mass (Figure 1B, Figure S1D). Consistent with these changes, muscle fibres were larger in EAA + RET (Figure 1C). Both RET and to a greater extent, EAA + RET increased muscle strength (both MCC and grip strength) compared with non‐RET groups (Figure 1D and Figure S1E). Muscle quality (i.e., muscle strength normalized to mass) was greater in both RET groups than in non‐RET groups (Figure 1E and Figure S1F). Interestingly, we found that EAA + RET increased endurance compared with CON and RET (Figure 1F). These results are the first demonstration that balanced free EAA supplementation during RET improved both muscle strength and endurance as compared with RET alone.

### Combined treatment of essential amino acids and resistance exercise training increases muscle mass through stimulating rate of muscle protein synthesis in part via activation of Akt1‐mTORC1 signalling and suppression of myostatin protein expression

**Figure 2:**
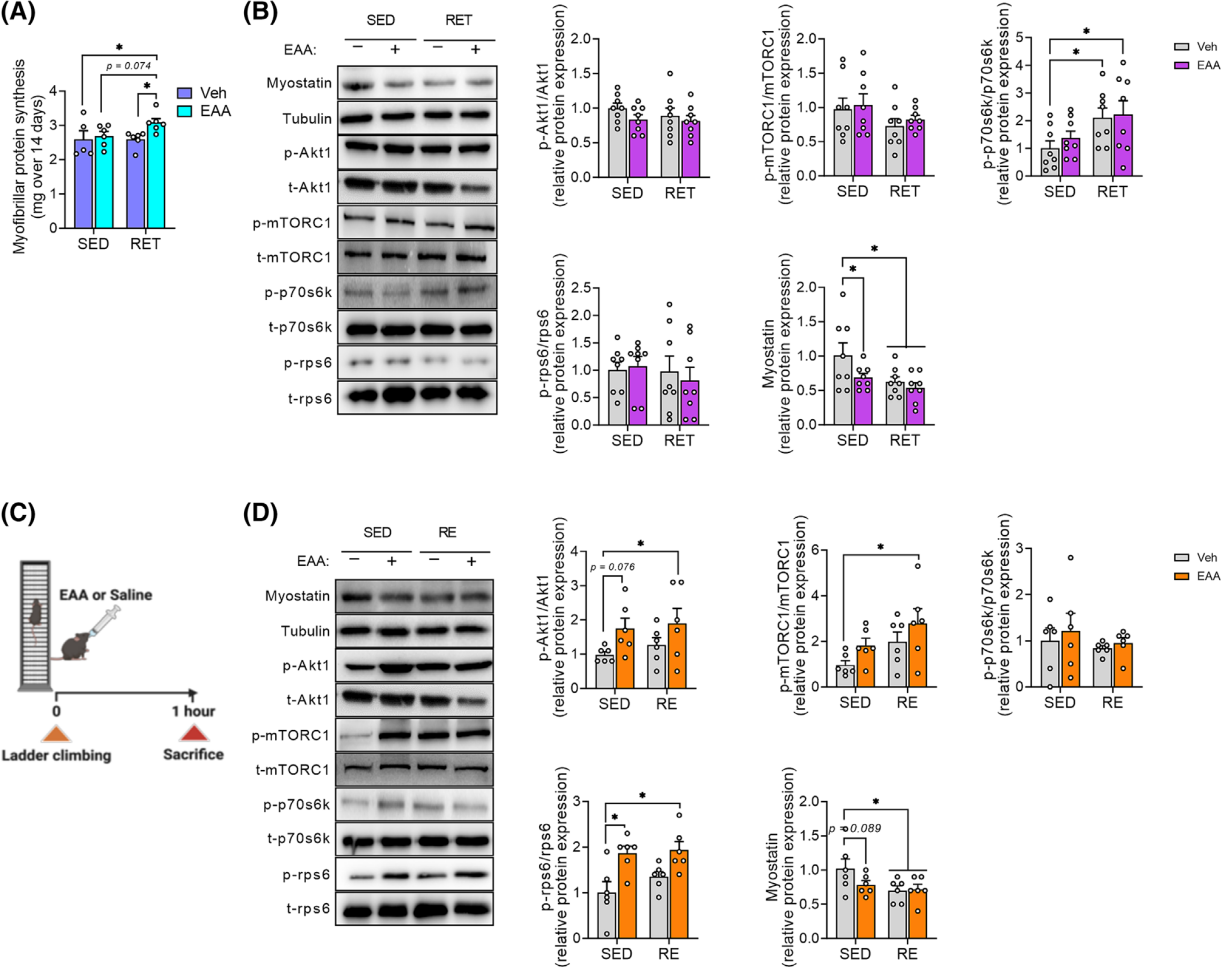
EAA + RET increases net muscle protein synthesis largely by stimulation of MPS via activation of Akt1/mTORC1 signalling and by suppression of MPB via suppression of myostatin expression. (A) Integrated myofibrillar protein synthesis rate in gastrocnemius over 14 days (*n* = 5–6 per group). (B) Chronic effect of EAAs and/or RET on relative protein expression of Akt1, mTORC1, p70s6k, rps6, and myostatin in gastrocnemius over 4 weeks and representative image (*n* = 8 per group). (C) Schematic representation of the experimental design: Mice were sacrificed 1 h after ladder climbing and/or oral EAA injection. This figure was created with BioRender.com. (D) Acute response to EAAs and/or resistance exercise on relative protein expression of Akt1, mTORC1, p70s6k, rps6, and myostatin in gastrocnemius and representative image (*n* = 6 per group). Data are presented as mean ± SE. *Significant difference between labelled groups (*P < 0.05). Akt1, protein kinase B; EAA, essential amino acids; mTORC1, mammalian target of rapamycin complex 1; myostatin, growth differentiation factor‐8; p70s6k, ribosomal protein S6 kinase beta‐1; RE, resistance exercise; RET, resistance exercise training; rps6, ribosomal protein s6; SED, sedentary; Veh, vehicle.

Muscle mass is tightly regulated by the balance between rates of MPS and MPB (muscle protein breakdown).^24^ To understand underlying metabolic mechanisms (i.e., protein kinetics), we quantified the cumulative rate of MPS in gastrocnemius. Consistent with increased muscle mass (Figure 1B), EAA + RET significantly increased rate of MPS, compared with other groups (Figure 2A).

Activation of Akt1/mTORC1 signalling is well known to play a vital role in stimulating MPS.^25^ EAAs, particularly leucine, can enhance the anabolic response by activation of mTORC1.^26^ Thus, we analysed this signalling pathway to assess chronic and acute effects of the respective treatments. For chronic response (4 weeks), we found no significant changes in Akt1, mTORC1, and rps6, except for increased p70s6k activity in RET or EAA + RET (Figure 2B), indicating the short‐lived nature of the effect of RET and/or EAA treatment. Therefore, we examined the activity of the Akt1/mTORC1 signalling pathway acutely (i.e., 1 h after the acute treatment of EAAs and/or RE) and found increased phosphorylation of Akt1, mTORC1, and rps6 compared with CON (Figure 2D).

To assess the role of the other side of muscle protein balance equation, we analysed factors implicated in MPB. We examined the expression of myostatin (growth differentiation factor‐8) as it is mainly expressed in skeletal muscle, which activates the transcription of muscle‐specific ubiquitin E3 ligase to increase protein breakdown and inhibits Akt1/mTORC1 signalling activity to decrease protein synthesis.^27^ We found that the expression of myostatin protein decreased (Figure 2B,D), but the mRNA expression E3 ligases (e.g., *Atrogin‐1* and *MuRF1*) did not change (Figure S2A,B) after both acute and chronic treatment.

### Resistance exercise training improves muscle quality through enhancement of neuromuscular junction stability

We measured ex vivo muscle contractile properties in isolated EDL to determine if the improved muscle quality in RET or EAA + RET was due to changes in intramuscular factors. However, we found no difference in the specific force and calcium sensitivity (Figure S3A–D) between treatments, indicating that changes in extrinsic factor(s) are responsible for the enhanced muscle quality.

**Figure 3:**
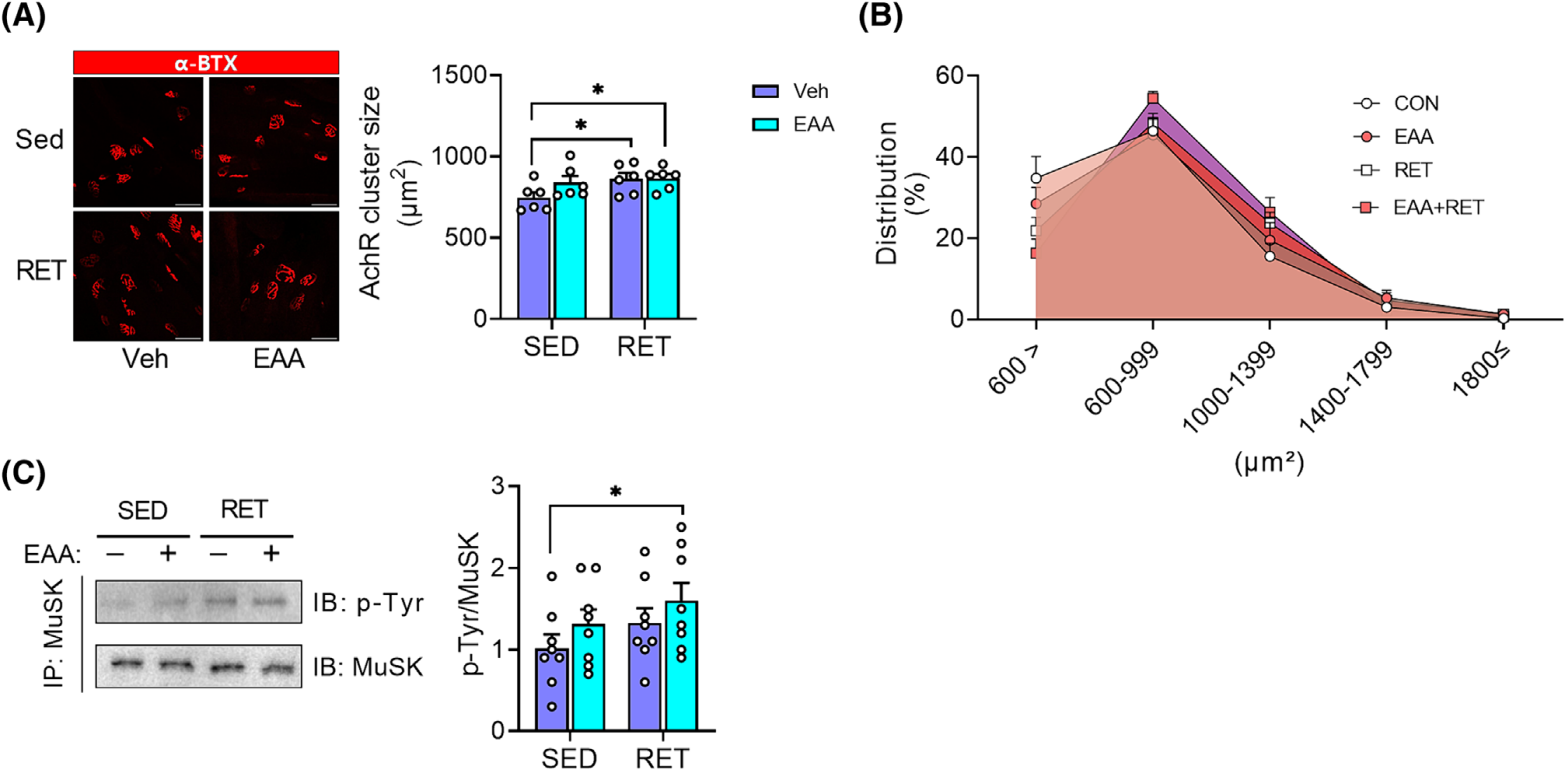
EAA + RET more robustly enhances neuromuscular junction stability. (A) Acetylcholine receptor cluster size in plantaris (*n* = 6 per group). Scale bar, 100 μm. (B) Acetylcholine receptor cluster size distribution. *(C)* Representative image of total and phosphorylated MuSK protein expression in plantaris (*n* = 8 per group). Data are presented as mean ± SE. *Significant difference between labelled groups (*P < 0.05). AchR, acetylcholine receptor; CON, control; EAA + RET, essential amino acids + resistance exercise training; EAA, essential amino acids; EAA, essential amino acids; MuSK, muscle‐specific tyrosine kinase; RET, resistance exercise training; SED, sedentary; Veh, vehicle; α‐BTX, alpha bungarotoxin.

Next, we investigated structural improvement of the NMJ as a potential mechanism of RET‐induced increase in muscle quality, because mutations in muscle‐specific tyrosine kinase (MuSK), which is required for the formation and maintenance of NMJ in mice, reduce muscle strength and is accompanied by severe musculoskeletal lesions.^28^ To this end, we analysed the morphology of the AchR and the activity of MuSK. In accordance with changes in muscle quality, RET and EAA + RET increase the AchR size compared with the CON (Figure 3A,B). In addition, EAA + RET increased MuSK activity compared with CON (Figure 3C). Furthermore, we found that AchR size was positively correlated with muscle strength or quality (Figure S4A–D). Therefore, these results suggest structural improvements in NMJ stability played a role in the improvements in muscle strength and quality in both RET and RET + EAA.

### Combined treatment of essential amino acids and resistance exercise training improves endurance capacity through enhanced rates of mitochondrial protein synthesis and dynamics

**Figure 4:**
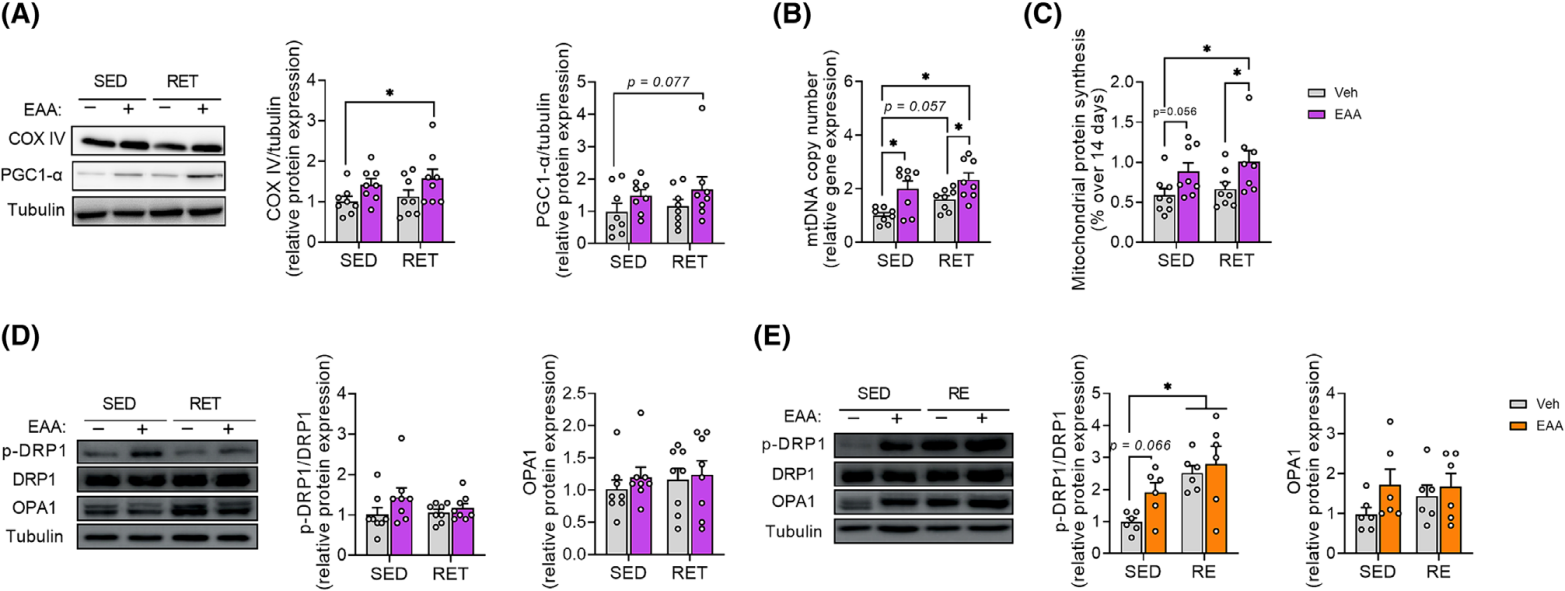
EAA treatment increases rate of mitochondrial protein synthesis and dynamics. (A) Western blots for COX IV and PGC‐1α in gastrocnemius and representative images (*n* = 8 per group). (B) The relative mtDNA copy number with qPCR measurement in gastrocnemius. (C) Integrative rate of mitochondrial protein synthesis over 14 days in gastrocnemius. (D) Chronic effect of EAAs and/or resistance exercise training on relative protein expression of DRP1 and OPA1 in gastrocnemius and representative image (*n* = 8 per group). (E) Acute response to EAAs and/or resistance exercise on relative protein expression of DRP1 and OPA1 in gastrocnemius and representative image (*n* = 6 per group). Data are presented as mean ± SE. *Significant difference between labelled groups (*P < 0.05). COX IV, cytochrome c oxidase subunit IV; DRP1, dynamin‐related protein 1; EAA, essential amino acids; GAS, gastrocnemius muscle; OPA1, dynamin‐like 120 kDa protein; PGC‐1α, peroxisome proliferator‐activated receptor‐gamma coactivator 1‐alpha; RE, resistance exercise; RET, resistance exercise training; SED, sedentary; Veh, vehicle.

We analysed factors related to mitochondrial biogenesis and protein synthesis to explore the mechanisms responsible for the improved endurance in the EAA + RET group. Consistent with increased endurance (Figure 1F), the level of COX IV (Figure 4A) protein and mtDNA copy number (Figure 4B) were most potently increased in EAA + RET, while PGC‐1α showed a trend compared with CON (Figure 4A). Moreover, the rate of mitochondrial protein synthesis was significantly increased in the muscles of the EAA + RET group compared with CON and RET groups, and the EAA‐only group showed an increased tendency compared with CON (Figure 4C). Consistent with these results, there were correlations between endurance and COX IV protein expression and mitochondrial protein synthesis (Figure S5B,C), albeit with a weak correlation with PGC‐1α (Figure S5A).

In addition, Mitochondrial fission and fusion are essential to maintain mitochondrial abundance and quality.^7^ Therefore, we examined the expression of proteins related to fission (DRP1) and fusion (OPA1) to determine whether the increased endurance was achieved at least in part, by enhancing mitochondrial dynamics. Similar to signalling responses implicated in muscle protein turnover, phosphorylation of DRP1 was acutely increased (not chronically) in response to EAAs or more potently EAA + RET but not OPA1 (Figure 4D,E). Our data therefore indicate that provision of balanced free EAAs during RET improves endurance by improving mitochondrial abundance and quality through stimulation of mitochondrial protein synthesis and regulation of mitochondrial dynamics, particularly the fission process.

### Essential amino acid treatment enhances mitochondrial oxidative capacity via an improvement of mitochondrial dynamics in DRP1‐dependent manner

**Figure 5:**
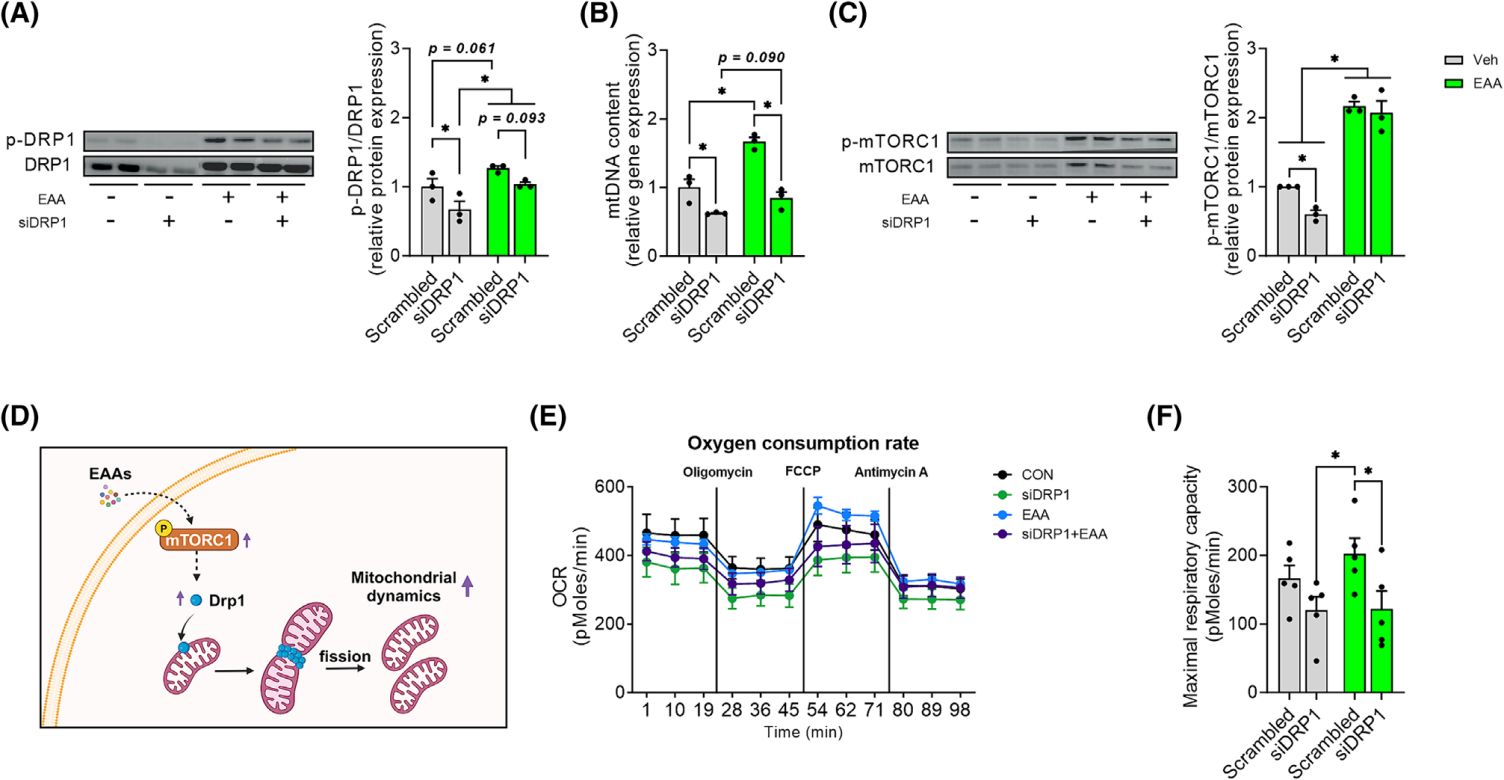
EAA treatment increases mitochondrial abundance and oxidative capacity in DRP1‐dependent manner. (A) Western blot for total and phosphorylated DRP1 after siRNA‐induced DRP1 knockdown and representative images (*n* = 3 per group). (B) The relative mtDNA copy number with qPCR measurement after siRNA‐induced DRP1 knockdown. (C) Western blot for total and phosphorylated mTORC1 after siRNA‐induced DRP1 knockdown and representative images. (D) Schematic representation of mTROC1‐dependent DRP1 activation by EAA supplementation. This figure was created with BioRender.com. (E) Oxygen consumption rate after siRNA‐induced DRP1 knockdown (*n* = 5 per group). (F) Maximal respirator capacity after siRNA‐induced DRP1 knockdown. Data are presented as mean ± SE. *Significant difference between labelled groups (*P < 0.05). CON, control; DRP1, dynamin‐related protein 1; EAA, essential amino acids; mTORC1, mammalian target of rapamycin complex 1; OCR, oxygen consumption rate; Veh, vehicle.

We knocked down DRP1 in C2C12 myotubes and then treated them with EAAs for 24 h to examine if free EAAs stimulate mitochondrial dynamics in DRP1‐dependent manner. We found that DRP1 knockdown (KD) decreased DRP1 protein levels as well as DRP1 activity whereas EAAs increased DRP1 activity. However, this increase was attenuated by DRP1 KD (Figure 5A). Similarly, DRP1 KD decreased mtDNA abundance whereas EAA treatment increased the abundance. Notably, the EAA‐induced increase in mtDNA abundance was decreased by DRP1 KD (Figure 5B). Interestingly, EAA treatment increased DRP1 protein expression in the presence of DRP1 KD (Figure 5A) which is probably due to enhanced protein translational efficiency via EAA‐mediated activation of mTORC1 activity as previously reported.^26^

It is known that mTORC1 is an upstream factor of DRP1.^9^ Thus, we examined if mTORC1 mediates the EAA‐induced activation of DRP1. Interestingly, we observed that DRP1 KD reduced the activation of mTORC1. However, EAA treatment blocked the DRP1 KD‐induced decrease in mTORC1 activity (Figure 5C). This response shows that activation of DRP1 by EAAs plays an important role in mitochondrial biogenesis in a mTORC1‐dependent manner.

Next, we explored whether the EAA‐mediated activation of mTORC1‐DRP1 axis leads to improvements in mitochondrial oxidative capacity. To do this, we analysed oxygen consumption rate (OCR). We found that DRP1 KD reduced both basal and maximal OCR, while EAA treatment increased maximal OCR (Figures 5E,F and S6A). However, this increase was completely blocked by DRP1 KD, indicating that EAA treatment increases mitochondrial function via activation of DRP1. In addition, EAA‐induced improvement of OCR disappeared when normalized to mtDNA contents (Figure S7A–C), indicating that the improvement was not due to changes in mitochondrial intrinsic activity (i.e., OCR normalized to mtDNA contents).

### Combined treatment of essential amino acids and resistance exercise training improves basal glucose metabolism

**Figure 6:**
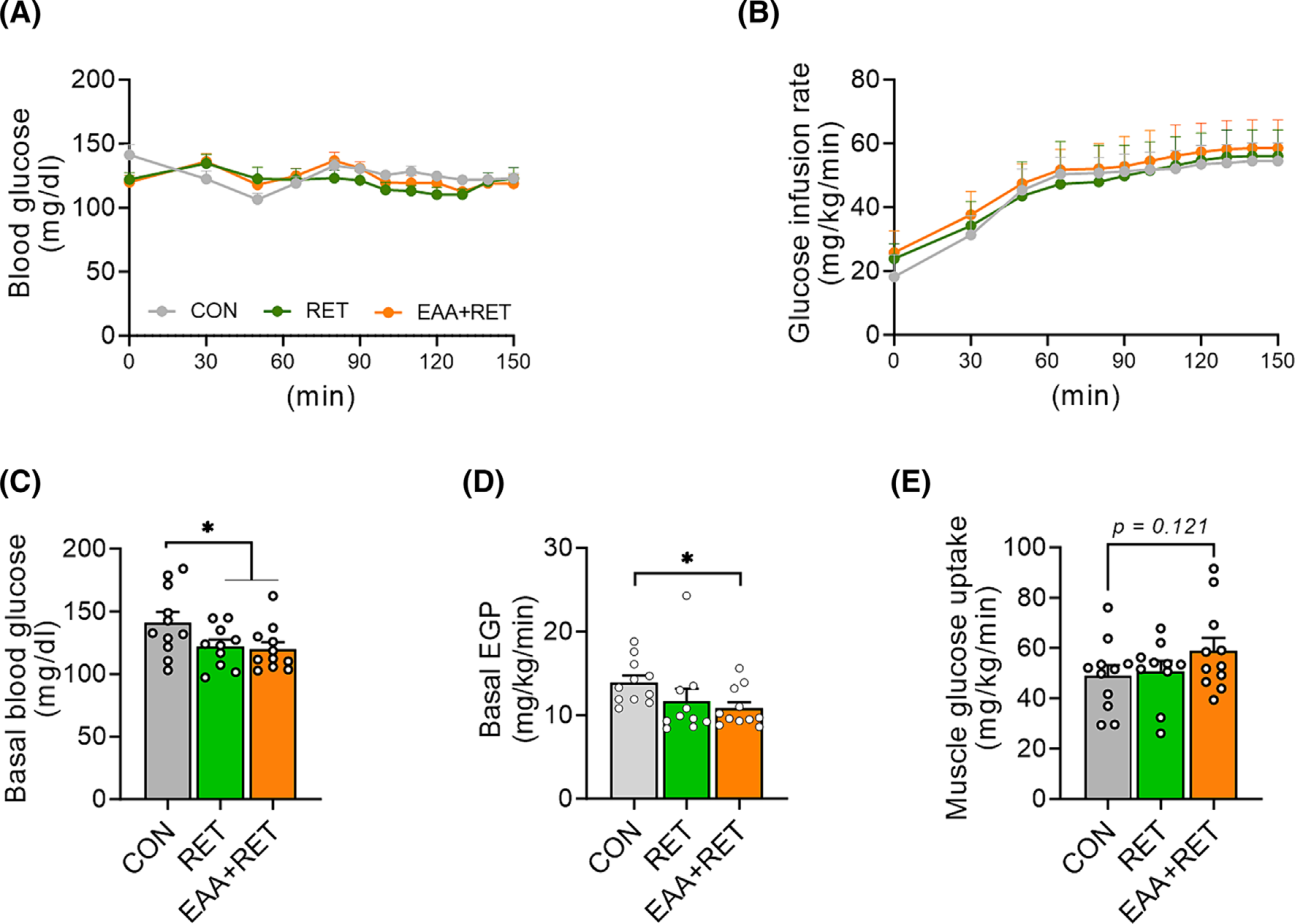
EAA + RET improves basal glucose kinetics. (A) Blood glucose concentration during hyperinsulinaemic–euglycaemic clamp (*n* = 10–11 per group). (B) Glucose infusion rate during the hyperinsulinaemic–euglycaemic clamp. (C) Basal blood glucose concentration. (D) Endogenous glucose production in basal state. (E) Muscle glucose uptake. Data are presented as mean ± SE. *Significant difference between labelled groups (*P < 0.05). CON, control; RET, resistance exercise training; EAA + RET, essential amino acids + resistance exercise training; EGP, endogenous glucose production.

Skeletal muscle plays a pivotal role in basal and insulin‐mediated glucose clearance^29^ and maintaining muscle mass is crucial for normal glucose metabolism. Therefore, we performed a hyperinsulinaemic–euglycaemic clamp to investigate whether gains in muscle mass and physical function by RET and EAAs have a positive effect on basal glucose metabolism and insulin sensitivity at the muscle and whole‐body levels. During the clamp, we observed no differences in the glucose infusion rates required to maintain euglycemia between the groups, indicating comparable whole‐body insulin sensitivities (Figures 6B and S8A,B). To better understand the contributions of liver and peripheral tissues to whole‐body insulin sensitivity, hepatic and peripheral (predominantly muscle) insulin sensitivities were measured separately during the clamp. We found no differences in hepatic and peripheral insulin sensitivities among groups (Figure S8C,D). However, consistent with a previous study,^30^ both RET groups exhibited reduced basal blood glucose concentration compared with the CON group (Figure 6A,C). The reduced basal glucose concentration was accomplished by a reduction in the rate of endogenous glucose production (EGP), primarily reflected by HGP (Figure 6D). Additionally, we observed a strong trend towards enhanced muscle glucose uptake in EAA + RET (Figure 6E).

## Discussion

Here we report that supplementation of free nine balanced EAAs during RET improves both muscle strength and endurance with concomitant improvements of basal glucose metabolism, suggesting the combination of balanced free EAA supplementation and RET as an effective muscle function booster, particularly for individuals with concerns about the detrimental effects of loss of muscle mass and function (e.g., sarcopenia).

RET is one of the most potent natural anabolic stimuli to gain muscle mass and strength by stimulating MPS. The maximal anabolic response requires adequate amino acid precursor availability. It has been found in both mice^31^ and humans^32^ that MPS exceeds MPB when RET is combined with increased amino acid availability. In line with this, our study revealed that RET‐induced gains in both relative and absolute muscle mass occurred only when combined with balanced free EAAs through an increased MPS. On the other hand, RET alone increased relative (% of body weight), but not absolute, muscle mass. The null effect of RET alone on absolute muscle mass may be due to the potential ‘aerobic’ nature of the ladder combing, although RET alone did not improve any endurance measures including endurance capacity and mitochondrial contents but improved muscle strength, indicating the dominance of the nature of the resistance training.

It is well known that at a molecular level, MPS is affected by the Akt1/mTORC1 axis, which can be activated by mechanical stimuli such as resistance exercise and/or amino acids, particularly leucine.^26^ However, actual increases in MPS cannot be achieved without proper provision of all nine EAAs.^33^ On the other side of protein balance equation, i.e., MPB, we noted no significant changes in factors among groups that affect MPB including ubiquitin‐proteasome system (UPS) (i.e., Atrogin‐1 and MuRF1), except for myostatin, a negative regulator of muscle mass, that exhibited reduced expression by the treatment of EAAs and/or RET. Taken together, our study demonstrated that supplementation of balanced nine free EAAs during RET amplified muscle mass gains partly through the stimulation of MPS via Akt1/mTORC1 signalling and the inhibition of myostatin expression.

We found that both RET and EAA + RET groups resulted in an improvement in muscle quality (muscle strength normalized to mass). The improvement in muscle quality observed in both RET groups in the current study must have arisen from changes in intramuscular and/or extra‐muscular factors. Single muscle cell fibre analysis revealed no differences in intrinsic contractibility of muscle isolated from mice, implying the important role of extra‐muscular factors. Instead, we identified that there was a significant positive correlation between muscle strength and NMJ stability. NMJ stability was significantly improved in both RET groups, as reflected by makers such as AchR clustering and MuSK activity. Our results are consistent with a previous study in which the loss of muscle strength was caused by congenital myasthenic syndromes.^34^ Moreover, increased MuSK activity, known as an essential regulator for the structural stability and function of AchR, has been reported to partially counteract muscle weakness caused by aging.^35^ Therefore, the improvement of muscle strength and quality by RET is attributed in part to the structural improvement of the NMJ rather than to the improvement of the intrinsic muscle fibre contractility.

Addition of dietary EAAs to RET not only improved muscle strength, but also improved endurance capacity. The gain in endurance capacity may have been related to improvements in two aspects of mitochondrial function: (1) mitochondrial fission and fusion and (2) mitochondrial protein turnover. Mitochondrial fission and fusion are essential for maintaining the abundance and quality of mitochondria.^7^ Mitochondrial fission is an important process for the abundance and quality of mitochondria, the latter of which can be achieved through selective removal of damaged components via mitophagy. DRP1 is an important mitochondrial fission factor, and loss of DRP1 is reported to be associated with a decline in mitochondrial function and muscle weakness.^36^ Conversely, increased expression of DRP1 in aged cells increases ATP production, which is directly related to exercise capacity and reduces ROS production.^37^ Dietary EAAs may up‐regulate DRP1 activity via its downstream effector, mTORC1, as it is reported that DRP1 activity.^9^ In line with this, we confirmed that the increase in mitochondrial content by balanced free EAA treatment in C2C12 cells was partially mediated by DRP1 activation.

Additionally, consistent with effects on mtDNA, we found that treatment with EAAs increased mTORC1 activity, which was not abolished by DRP1 knockdown, confirming that mTORC1 is an upstream factor regulating DRP1. However, we also found that DRP1 knockdown inhibited mTORC1 activation in the absence of EAAs, implying that a feedback loop exists between the two factors (i.e., mTORC1 and DRP1). We also confirmed that balanced free EAA treatment affects mitochondrial function (i.e., maximal oxygen consumption rate) in a DRP1‐dependent manner. Thus, it is likely that increased endurance by balanced free EAA supplementation during RET is due in part to increased mitochondrial number and quality improvement through increased mitochondrial protein synthesis and mitochondrial fission mediated by increased activity of mTORC1‐DRP1 signalling axis, ultimately leading to the enhancement of mitochondrial function (represented by maximal respiratory capacity and reserve capacity).

Skeletal muscle not only plays a crucial role in physical function but also influences health and disease processes. It is the most important organ for regulating postprandial glucose disposal, accounting for ~80% of peripheral glucose disposal.^38^ Thus, it is expected that increased muscle mass and/or function are expected to improve glucose metabolism. Recent studies provided direct evidence of the beneficial effect of increased muscle mass on glucose metabolism.^39^, ^40^ For instance, myostatin (GDF8) homozygous knockout doubled skeletal muscle mass and improved glucose tolerance and insulin sensitivity.^40^ Further, muscle‐specific overexpression of Akt1, inducing about 40% muscle growth, reduced insulin resistance.^39^ However, we did not observe an increase in whole‐body insulin sensitivity, including skeletal muscle glucose uptake. This discrepancy occurred due likely to the fact that both RET groups exhibited only a modest increase in skeletal muscle mass, which may not have been sufficient to significantly impact insulin sensitivity, particularly because the mice were not insulin‐resistant to begin with. Nevertheless, we observed the most robust reduction in basal plasma glucose concentrations with the combined treatment of balanced free EAAs and RET, which was accomplished primarily by a decrease in the EGP. Taken together, our findings suggest that balanced EAA supplementation during RET can facilitate the beneficial impact of exercise on basal glucose metabolism.

In summary, our study demonstrated that balanced free EAA supplementation during RET is an effective approach to induce a simultaneous improvement of both muscle strength and endurance in part through increased muscle mass with improvement of NMJ stability and dynamic remodelling of mitochondrial architecture in a DRP1‐dependent manner, leading in turn to improved basal glucose metabolism. Our findings may provide valuable insight into discovering effective treatments for muscle wasting such as sarcopenia, but because no females were utilized in this study, the results may be limited to only males. Further investigation is required to confirm these results in humans. Finally, it will be interesting to investigate if supplementation with balanced free EAAs during endurance training can lead to an improvement of muscle mass and/or strength in addition to an improvement in endurance.

## Funding

This research was funded by the National Research Foundation of Korea (NRF) grant funded by the Korean government (MSIT) (No. 2021R1A2C3005801). This research was also supported by the Bio & Medical Technology Development Program of the National Research Foundation (NRF) funded by the Korean government (MSIT) (No. 2014M3A9D5A01073886), Korea Health Technology R&D Project through the Korea Health Industry Development Institute (KHIDI) funded by the Ministry for Health and Welfare of Korea (No. HR14C0001), Ministry of Education of the Republic of Korea, National Research Foundation of Korea (NRF) (No. 2022S1A5C2A03092407), and American Heart Association Career Development Award (No. 23CDA1046498).

## Conflict of interest

I.‐Y.K. and S.P. are stockholders of Myocare. Inc., and R.R.W is a shareholder in the Amino Company, LLC., and holds patents on several essential amino acid‐based compositions, and serves as an advisor to Myocare, Inc. S.S. provides consulting and collaborative research studies to the Leducq Foundation (CURE‐PLAN), Red Saree Inc., Greater Cincinnati Tamil Sangam, Affinia Therapeutics Inc., Cosmogene Skincare Private Limited, Amgen and AstraZeneca, but such work is unrelated to the content of this article. All others have no potential conflict of interest.

## Supporting information


**Figure S1.** RET improves muscle strength and muscle quality regardless of EAA supplementation
**Figure S2.** EAAs and/or resistance exercise did not affect E3 ligases gene expression in both chronic and acute treatments.
**Figure S3.** The increase in muscle quality by RET was not due to the improvement of the intrinsic contractile property of muscle.
**Figure S4.** Muscle strength and quality are positively correlated with AchR cluster size.
**Figure S5.** EAA supplementation‐induced improvement of endurance capacity is positively correlated with mitochondrial abundance and rate of mitochondrial protein synthesis.
**Figure S6.** DRP1 knockdown decreases basal respiration of mitochondria.
**Figure S7.** EAA‐induced improvement of oxygen consumption rate is due to an increase in mitochondrial abundance. (A) Maximal respiratory capacity normalized by mtDNA contents (*n* = 5 per group). (B) Basal respiration normalized by mtDNA contents. (C) Reserve capacity normalized by mtDNA contents. Data are presented as mean ± S.E. *Significant difference between labelled groups (*p < 0.05). Veh, Vehicle; EAA, Essential amino acids; OCR, Oxygen consumption rate.
**Figure S8.** RET and/or EAA does not affect insulin‐stimulated glucose metabolism. (A) Area under the curve of glucose concentration for 150 mins (*n* = 10–11 per group). (B) Area under the curve of glucose infusion rate for 150 mins. (C)

**Table S1** Essential amino acids composition
**Table S2** List of antibodies
**Table S3** Primer sequence
**Table S4** siRNA target sequence
