# Supplementary material for: Free essential amino acid feeding improves endurance during resistance training via DRP1‐dependent mitochondrial remodelling
Source: J Cachexia Sarcopenia Muscle. 2024 Jun 16;15(5):1651–63. doi: 10.1002/jcsm.13519 (PMC11446676; doi:10.1002/jcsm.13519)
Supplement: Supplementary file 1 — Figure S1. RET improves muscle strength and muscle quality regardless of EAA supplementation Figure S2. EAAs and/or resistance exercise did not affect E3 ligases gene expression in both chronic and acute treatments. Figure S3. The increase in muscle quality by RET was not due to the improvement of the intrinsic contractile property of muscle. Figure S4. Muscle strength and quality are positively correlated with AchR cluster size. Figure S5. EAA supplementation‐induced improvement of endurance capacity is positively correlated with mitochondrial abundance and rate of mitochondrial protein synthesis. Figure S6. DRP1 knockdown decreases basal respiration of mitochondria. Figure S7. EAA‐induced improvement of oxygen consumption rate is due to an increase in mitochondrial abundance. (A) Maximal respiratory capacity normalized by mtDNA contents (n = 5 per group). (B) Basal respiration normalized by mtDNA contents. (C) Reserve capacity normalized by mtDNA contents. Data are presented as mean ± S.E. *Significant difference between labelled groups (*p < 0.05). Veh, Vehicle; EAA, Essential amino acids; OCR, Oxygen consumption rate. Figure S8. RET and/or EAA does not affect insulin‐stimulated glucose metabolism. (A) Area under the curve of glucose concentration for 150 mins (n = 10–11 per group). (B) Area under the curve of glucose infusion rate for 150 mins. (C) [file JCSM-15-1651-s002.docx]

***
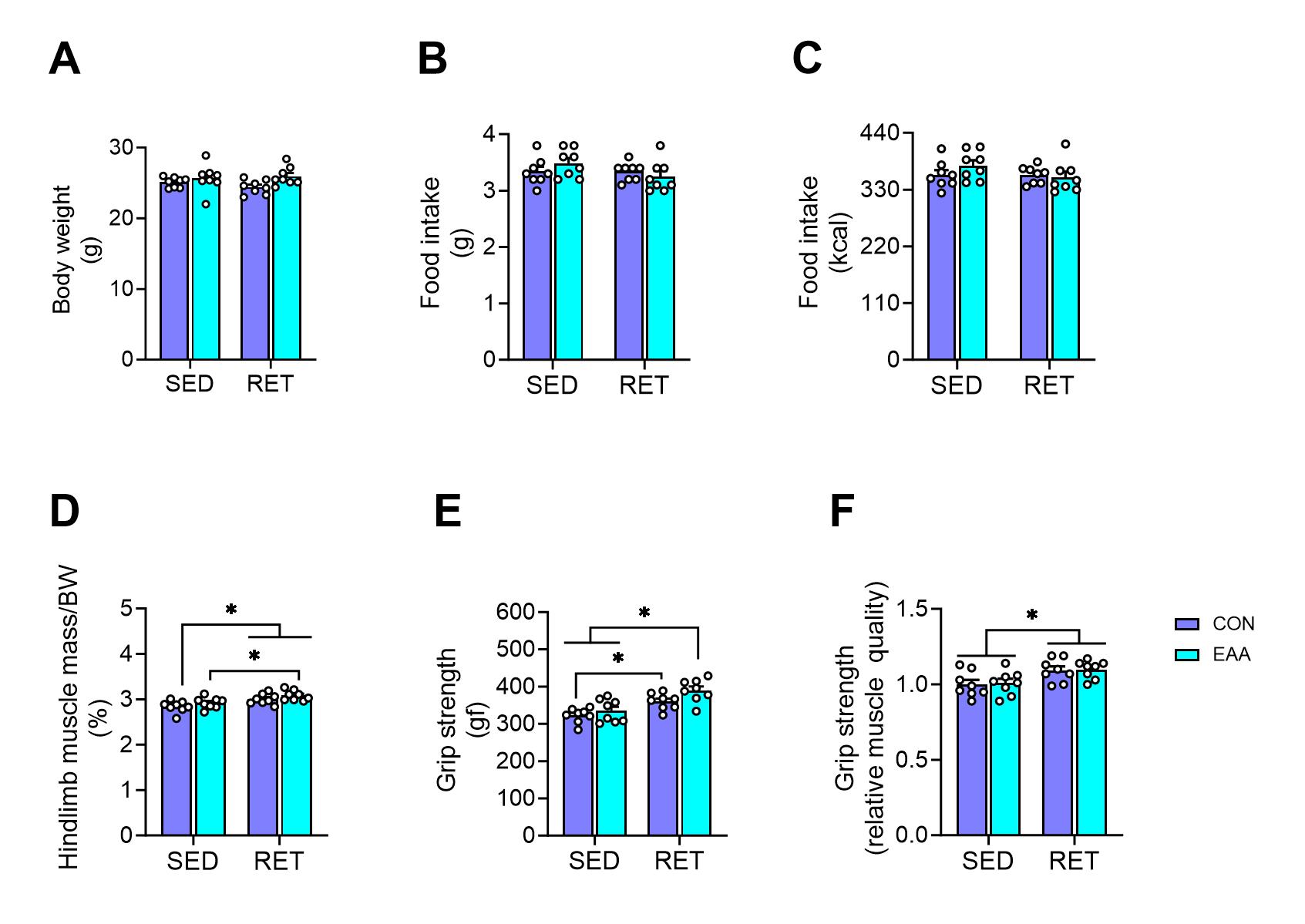
***

**Supplementary Figure 1. RET improves muscle strength and muscle quality regardless of EAA supplementation.**

(A) Final body weight (n = 8 per group). (B) Average food intake over 4 weeks. **(C) Total calory intake over 4 weeks. (D) Relative muscle mass (%).** (**E**) Grip strength. (**F**) Relative muscle quality in grip strength (grip strength normalized by hindlimb muscle mass). Data are presented as mean ± S.E. *Significant difference between labeled groups (****p*** < 0.05). Veh, Vehicle; SED, Sedentary; RET, Resistance exercise training; EAA, Essential amino acids.


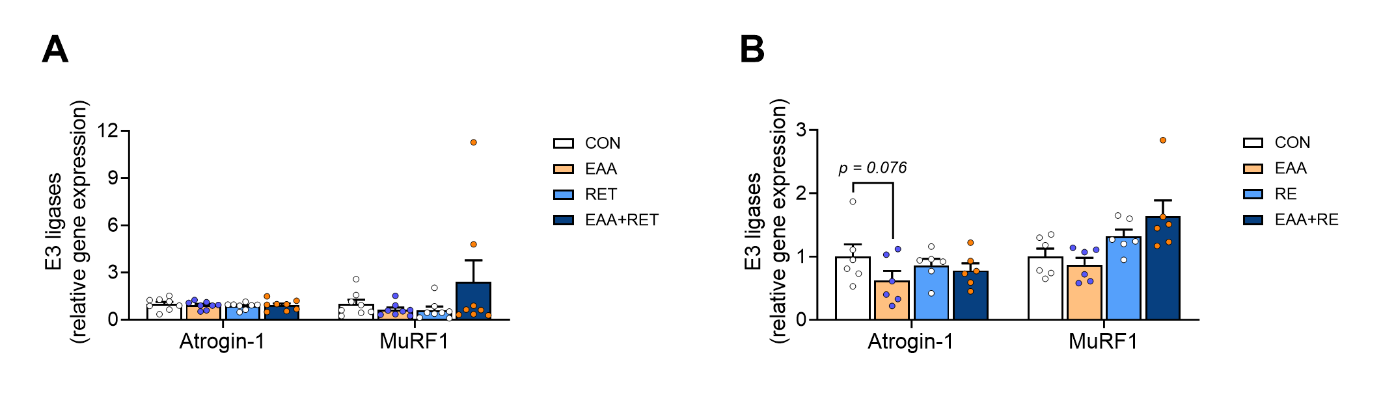
 **Supplementary Figure 2. EAA and/or resistance exercise did not affect E3 ligases gene expression in both chronic and acute treatments.**

(A) Chronic effect of EAA and/or resistance exercise training on relative gene expression of ***Atrogin-1*** and ***MuRF1*** (n = 8 per group). (B) Acute response to EAA and/or resistance exercise on relative gene expression of ***Atrogin-1*** and ***MuRF1*** (n = 6 per group). Data are presented as mean ± S.E. CON, Control; EAA, Essential amino acids; RET, Resistance exercise training; RE, Resistance exercise; EAA + RET, Essential amino acids + resistance exercise training; EAA + RE, Essential amino acids + resistance exercise; ***Atrogin-1***, Muscle atrophy F-box protein; ***MuRF1***, Muscle ring finger protein-1.


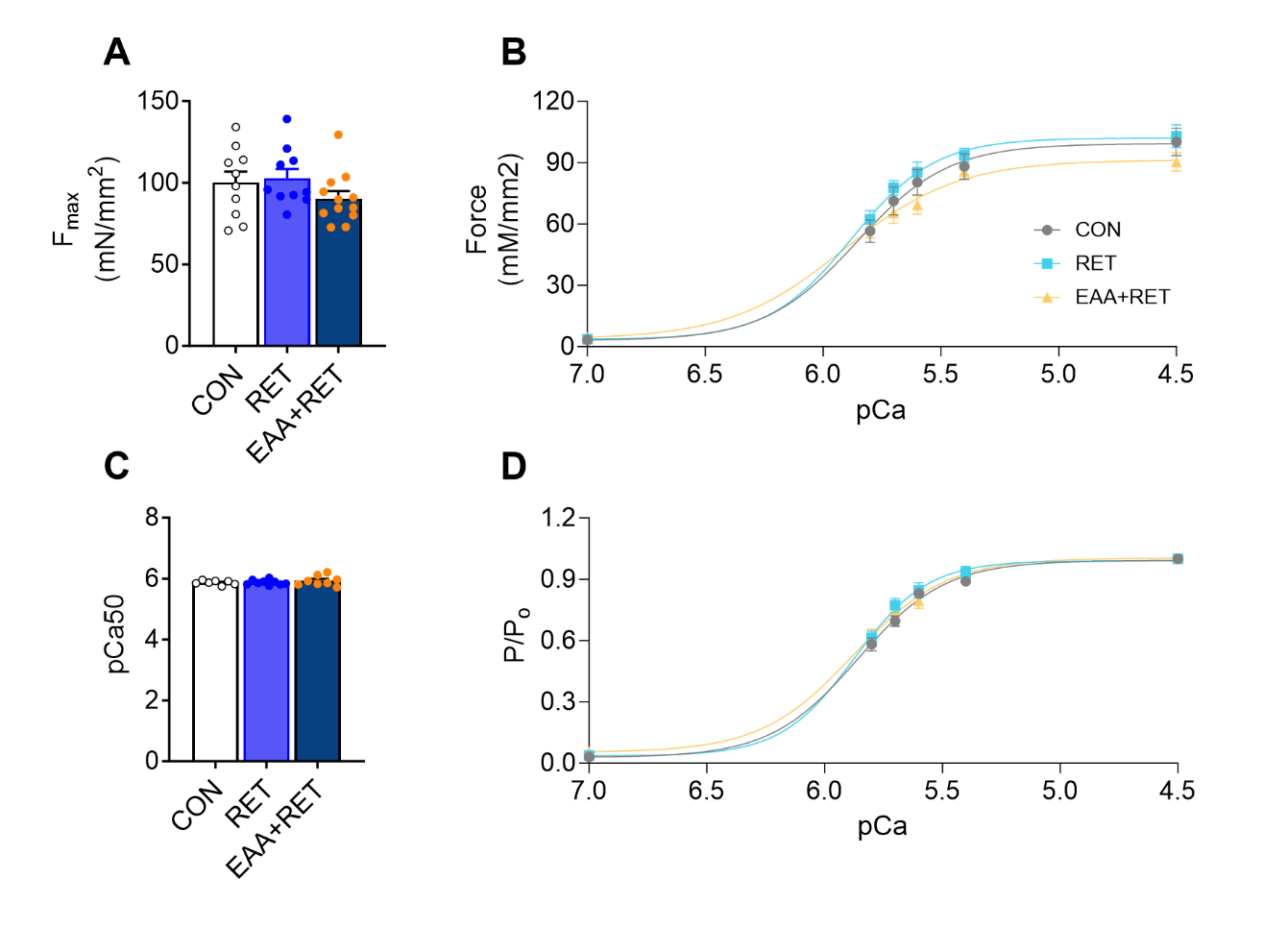
 **Supplementary Figure 3. The increase in muscle quality by RET was not due to the improvement of the intrinsic contractile property of muscle.**

(A) Maximum isometric force at pCa 4.5 (n = 10–12 per group). (B) Force–pCa curve of skinned single EDL fiber in pCa 7.0 to 4.5 at 2.3 μm sarcomere length. (C) Myofilament calcium sensitivity of contraction (pCa50). (D) Force relative to isometric force. Data are presented as mean ± S.E. CON, control; RET, Resistance exercise training; EAA + RET, Essential amino acids+ Resistance exercise training; F_max_, Maximum isometric force; pCa50, Calcium sensitivity; P/P_0_, Relative isometric force.


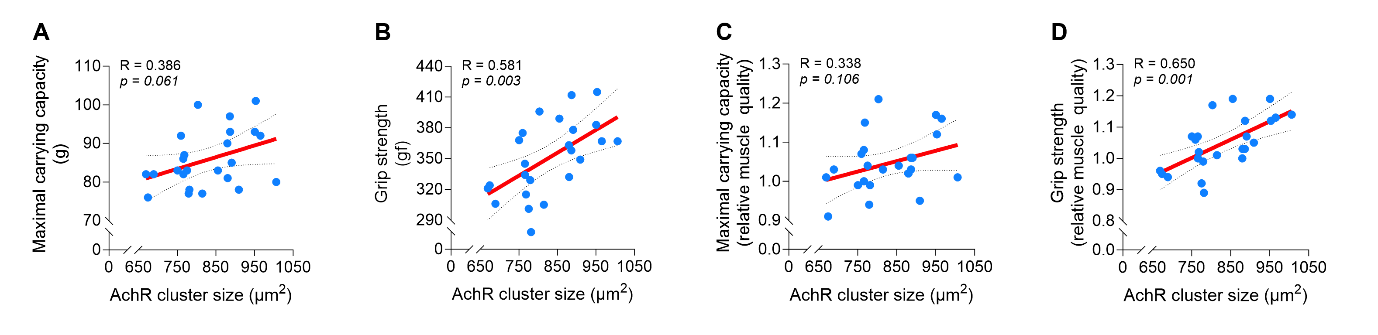
 **Supplementary Figure 4. Muscle strength and quality are positively correlated with AchR cluster size.**

(A) Correlation between AchR cluster size and maximal carrying capacity. (B) Correlation between AchR cluster size and grip strength. (C) Correlation between AchR cluster size and relative muscle quality of maximal carrying capacity. (D) Correlation between AchR cluster size and relative muscle quality of grip strength. Data are presented as mean ± S.E. AchR, Acetylcholine receptor.


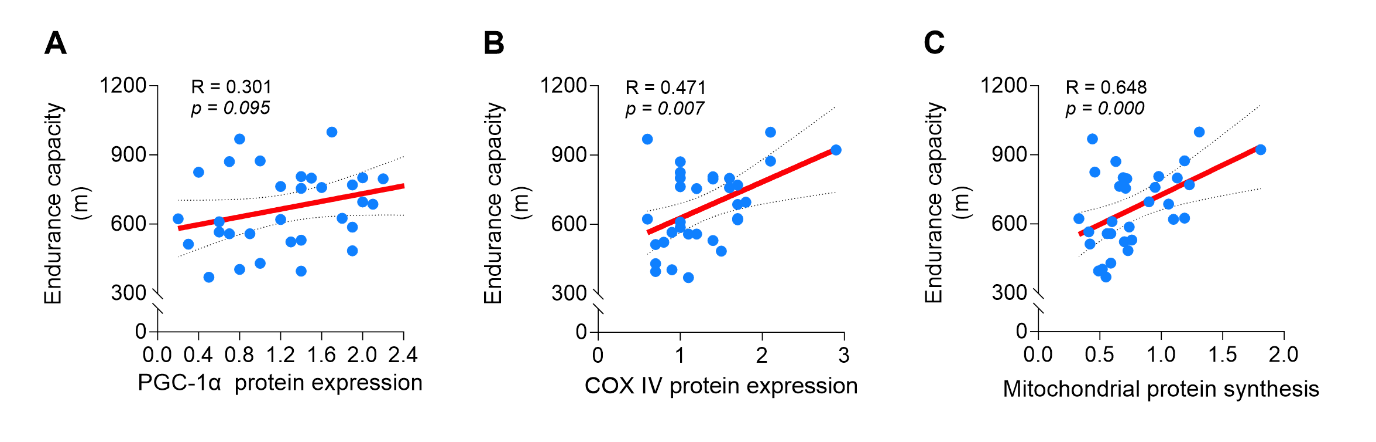
 **Supplementary Figure 5. EAA supplementation-induced improvement of endurance capacity is positively correlated with mitochondrial abundance and rate of mitochondrial protein synthesis.**

(A) Correlation between PGC-1α protein expression and endurance capacity. (B) Correlation between COX IV protein expression and endurance capacity. (C) Correlation between mitochondrial protein synthesis and endurance capacity. Data are presented as mean ± S.E. PGC-1α, Peroxisome proliferator-activated receptor-gamma coactivator 1-alpha; COX IV, Cytochrome c oxidase subunit IV.


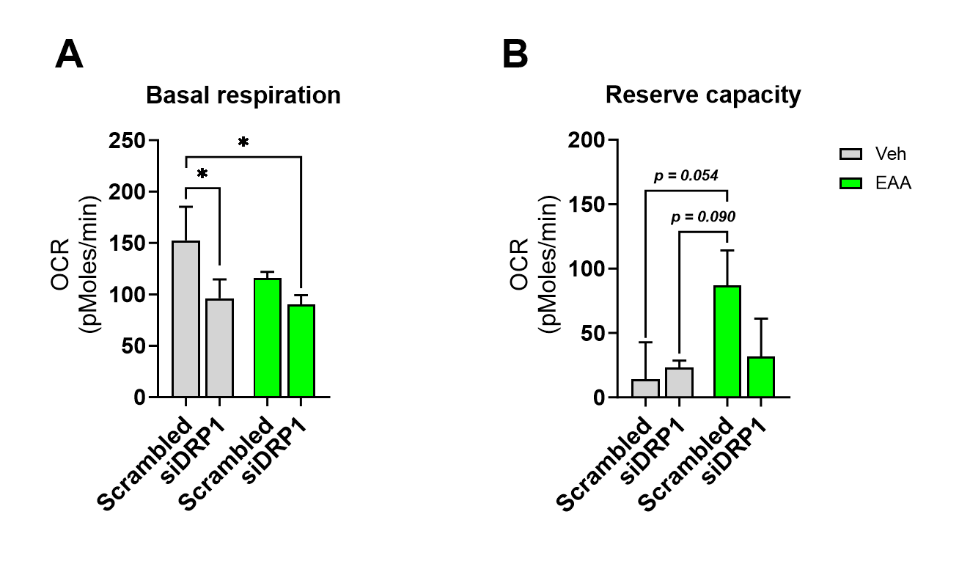
 **Supplementary Figure 6. DRP1 knockdown decreases basal respiration of mitochondria.**

(A) Basal respiration after siRNA induced DRP1 knockdown (n = 5 per group). (B) Reserve capacity after siRNA induced DRP1 knockdown. Data are presented as mean ± S.E. *Significant difference between labeled groups (****p*** < 0.05). Veh, Vehicle; EAA, Essential amino acids; OCR, Oxygen consumption rate.


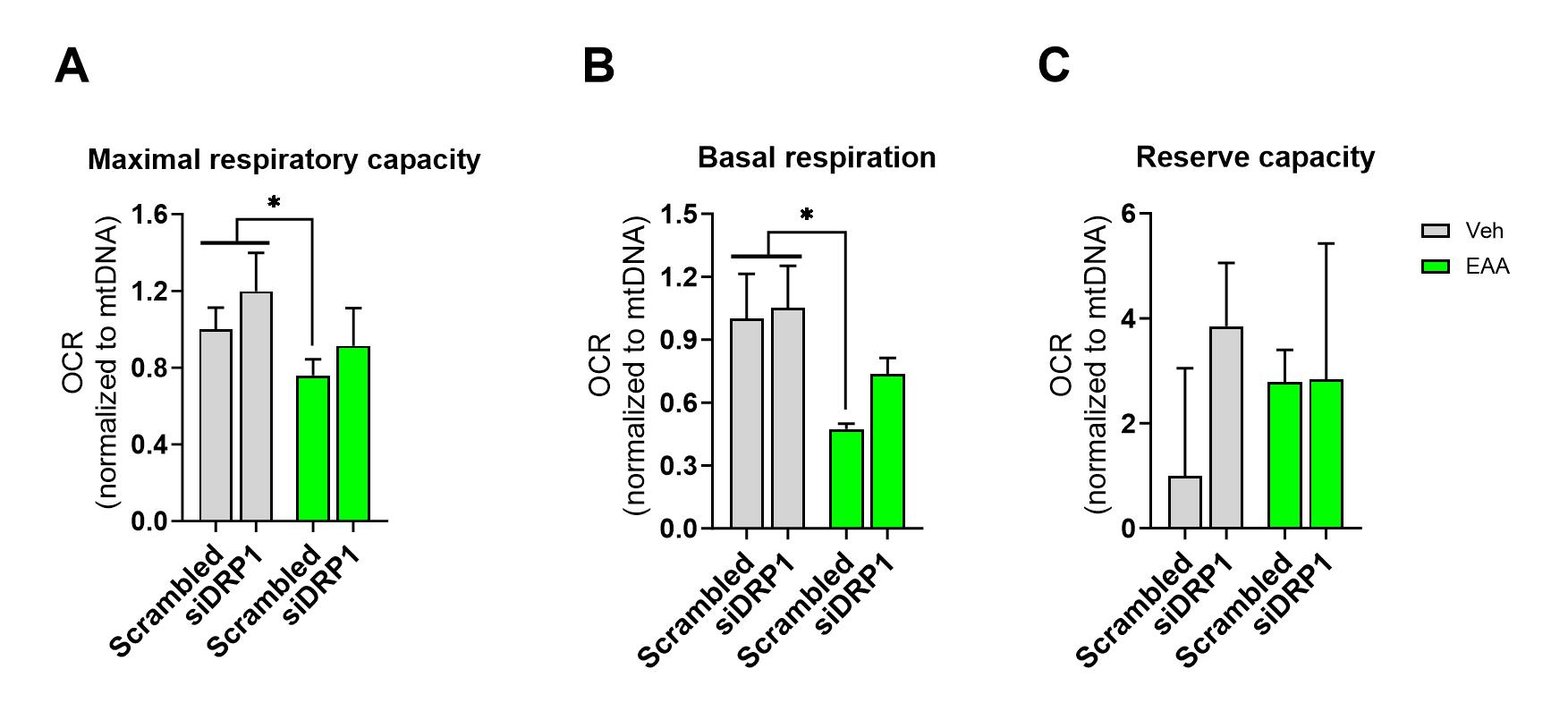


**Supplementary Figure 7. EAA-induced improvement of oxygen consumption rate is due to an increase in mitochondrial abundance.**

**(A) Maximal respiratory capacity normalized by mtDNA contents (n = 5 per group). (B) Basal respiration normalized by mtDNA contents. (C) Reserve capacity normalized by mtDNA contents. Data are presented as mean ± S.E. *Significant difference between labeled groups (**p* < 0.05). Veh, Vehicle; EAA, Essential amino acids; OCR, Oxygen consumption rate.**

***
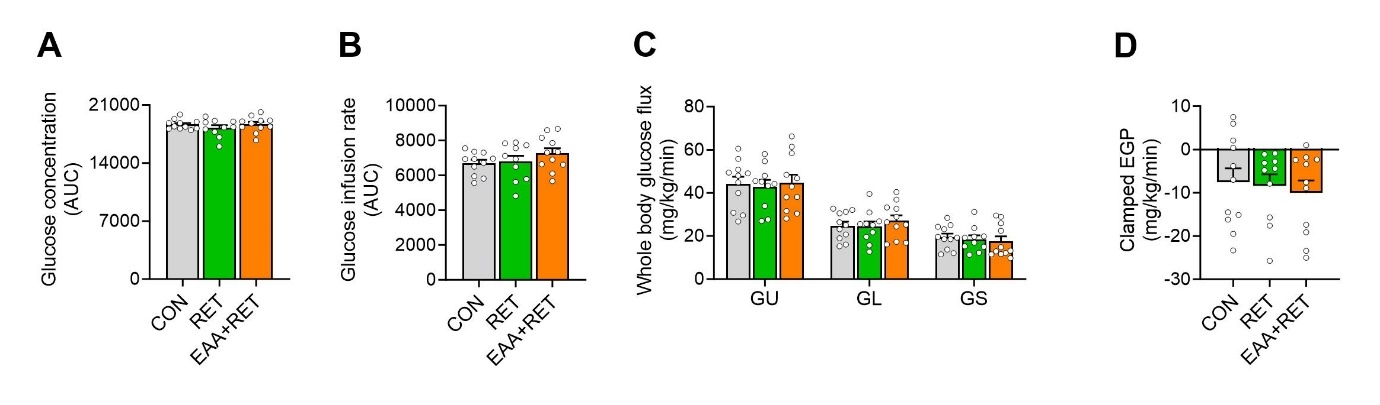
* Supplementary Figure 8. RET and/or EAA does not affect insulin-stimulated glucose metabolism.**

**(A) Area under the curve of glucose concentration for 150 mins (n = 10-11 per group). (B)** **Area under the curve** **of glucose infusion rate for 150 mins.** **(C)** Whole-body glucose flux during the clamp. **(D)** Endogenous glucose production during the clamp. Data are presented as mean ± S.E. CON, Control; RET, Resistance exercise training; EAA + RET, Essential amino acids + Resistance exercise training; EGP, Endogenous glucose production; GU, Glucose uptake; GL, Glycolysis; GS, Glycogen synthesis.
