# Supplementary material for: Free essential amino acid feeding improves endurance during resistance training via DRP1‐dependent mitochondrial remodelling
Source: J Cachexia Sarcopenia Muscle. 2024 Jun 16;15(5):1651–63. doi: 10.1002/jcsm.13519 (PMC11446676; doi:10.1002/jcsm.13519)
Supplement: Supplementary file 2 — Table S1 Essential amino acids composition Table S2 List of antibodies Table S3 Primer sequence Table S4 siRNA target sequence [file JCSM-15-1651-s001.docx]

| **Amino Acid** | **Percentage (%)** | **Dietary Intake (g/kg/day)** |
| --- | --- | --- |
| Histidine | 10 | 0.150 |
| Isoleucine | 10 | 0.150 |
| Leucine | 21 | 0.315 |
| Lysine | 18 | 0.270 |
| Methionine | 4 | 0.060 |
| Phenylalanine | 12 | 0.180 |
| Threonine | 14 | 0.210 |
| Valine | 10 | 0.150 |
| Tryptophan | 1 | 0.015 |
| **Total** | **100** | **1.5** |

**Table S1 Essential amino acids composition**

**Table S2 List of antibodies**

| **Antibodies** | **Source** | **Identifier** |
| --- | --- | --- |
| Akt1 | Cell signaling technology | #9272 |
| p-Atk1 (Ser473) | Cell signaling technology | #9271 |
| mTORC1 | Cell signaling technology | #2983 |
| p-mTORC1 (Ser2448) | Cell signaling technology | #2971 |
| p70s6k | Cell signaling technology | #9202 |
| p- p70s6k (Ser371) | Cell signaling technology | #9208 |
| rps6 | Cell signaling technology | #9272 |
| p-rps6 (Ser473) | Cell signaling technology | #9271 |
| COX IV | Cell signaling technology | #4844S |
| DRP1 | Cell signaling technology | #8570S |
| p-DRP1 (Ser616) | Cell signaling technology | #4494 |
| Anti-rabbit IgG | Cell signaling technology | #7074 |
| Anti-mouse IgG | Cell signaling technology | #7076 |
| rabbit anti-laminin | Cell signaling technology | #L9393 |
| PGC-1α | Abcam | ab54481 |
| Myostatin | Abcam | ab71808 |
| MuSK | Santa cruz Biotechnology | sc-134398 |
| p-Tyr | Santa cruz Biotechnology | sc-508 |
| OPA1 | BD Biosciences | 612606 |
| β-tubulin | Merck Millipore | #05-661 |
| Alexa Fluor 488-conjugated anti-rabbit IgG | Invitrogen | #A-21121 |
| Alexa Fluor 488-conjugated anti-rabbit α-Bungarotoxin | Thermo Fisher | B13422 |

**Table S3 Primer sequence**

| **Primer** | **Forward** | **Reverse** |
| --- | --- | --- |
| mtMDA4 | CTCCTCAGTTAGCCACATAGCA | TGTGGATCCGTTCGTAGTTGGA |
| 28S rRNA | AGGACCCGAAAGATGGTGAACTA | CGGAGGGAACCAGCTACTAGAT |
| Atrogin-1 | GACTGGACTTCTCGACTGCC | TCAGGGATGTGAGCTGTGAC |
| MuRF1 | AAGCAGGTGCCACTCTCTGT | AGCTTCACACCTGTCCTTCG |
| GAPDH | CACCATCTTCCAGGAGCGAG | CCTTCTCCATGGTGGTGAAGAC |

**Table S4 siRNA target sequence**

| **siRNA** | **Target sequence** |
| --- | --- |
| DRP1 | UUCAAUCCGUGAUGAGUAUGCUUUUCUUCUU |
| Control | CCUCGUGCCGUUCCAUCAGGUAGUU |
